# 6‐Monoacetylmorphine‐antibody distribution in tissues from heroin‐related death cases: An experimental study to investigate the distributive response

**DOI:** 10.1111/jcmm.17351

**Published:** 2022-08-02

**Authors:** Aniello Maiese, Raffaele La Russa, Maria Chiara David, Santina Cantatore, Alice Chiara Manetti, Alessandra De Matteis, Costantino Ciallella, Paola Frati, Vittorio Fineschi

**Affiliations:** ^1^ Institute of Legal Medicine Department of Surgical Pathology Medical, Molecular and Critical Area University of Pisa Ospedale Santa Chiara Pisa Italy; ^2^ Section of Legal Medicine Department of Clinical and Experimental Medicine University of Foggia Ospedale Colonnello D'Avanzo Foggia Italy; ^3^ Department of Public Security Health Central Directorate Research Center and Forensic Toxicology Laboratory Ministry of the Interior Rome Italy; ^4^ Department of Anatomical, Histological, Forensic and Orthopaedic Sciences Sapienza University of Rome Rome Italy

**Keywords:** anti‐6‐MAM antibody, heroin‐related death, immunohistochemistry, marker, post‐mortem diagnosis

## Abstract

Heroin, a semisynthetic opioid drug synthesized from morphine, is the 3,6‐diacetyl ester of morphine (diacetylmorphine). The post‐mortem diagnosis of heroin‐related death could be an issue and usually rely on a combination of investigations, including the autopsy, histological and toxicological analysis. We conducted the present study to evaluate the correlation between the heroin concentration in biological fluids (peripheral blood, bile and urine) and the post‐mortem anti‐6‐MAM antibody expression in various tissues (brain, heart, lung, liver and kidney) using immunohistochemical staining. A quantitative analysis of the immunohistochemical reaction was carried out. 45 cases of heroin‐related death investigated at the Forensic Pathology Institutes of the University of Rome, Foggia and Pisa were included. The control group was composed of 15 cases of death due to other causes, without brain lesions and negative toxicological analysis for drugs. We found a positive immunohistochemical reaction in different organs and it was related to the timing of heroin metabolization. No reaction was found in the control group. Our findings show that immunohistochemistry can be a valuable tool for the post‐mortem diagnosis of acute heroin abuse. A better understanding of the timing of heroin's metabolism can be useful in the forensic field and for future therapeutic applications.

## 1 INTRODUCTION

Heroin, a semisynthetic opioid drug synthesized from morphine, is the 3,6‐diacetyl ester of morphine (diacetylmorphine). Commercial heroin is diluted with sugars and adulterated with local anaesthetics, amphetamine‐like substances, cocaine and caffeine.^1^, ^2^ Worldwide, about 0.5 million deaths are attributable to drug use. More than 70% of these deaths are related to opioids, with more than 30% of those deaths caused by overdose.^3^ In Europe, in 2019, the average retail purity of heroin ranged from 11% to 51%, with half of the countries reporting an average purity between 18% and 31%. Indexed trends show that the average purity of heroin increased by 23% between 2009 and 2019, while its price decreased by 17%. National prevalence estimates range from less than one to more than seven high‐risk opioid users per 1.000 inhabitants aged 15–64 years. Overall, this translates into about 0.35% of the European population or 1 million high‐risk opioid users in 2019. Heroin was the third most common drug reported by Euro‐DEN Plus hospitals in 2019, present in 16% of hospital admissions for acute drug‐related toxicity. Opiates were found in 10 of the 26 hospital deaths reported, usually in combination with other drugs.^4^ The peak concentrations in the blood are generally at 1–5 min from an intravenous injection and 5 min after snorting or intramuscular administration. Heroin is 2–3 times more potent than morphine and the estimated minimum lethal dose is 100–200 mg, but addicts may be able to tolerate up to 10 times as much. However, fatalities have occurred after doses of 10 mg. Compared with morphine, heroin is a more lipophilic compound and crosses the blood–brain barrier within 15–20 s and achieves relatively high brain levels; 68% of an intravenous dose is absorbed into the brain.^5^ Heroin (diacetylmorphine) is rapidly transformed into its active metabolites (i.e., 6‐monoacetylmorphine [6‐MAM], morphine, morphine‐3‐glucuronide [M3G] and morphine‐6‐glucuronide [M6G]), primarily in peripheral blood and to some extent in the liver, kidney and brain^6^; with studies showing that the narcotic effects of heroin occur primarily via its major metabolite, 6‐MAM.^7^ Heroin and 6‐MAM are highly lipophilic, easily crossing the blood–brain barrier (BBB), yet they are rapidly metabolized to opiate agonists (i.e., morphine and M6G) and the likely neurotoxic M3G.^8^ In addition, the maximal brain concentrations (T_max_) of 6‐MAM were achieved at 15 min after heroin administration, similar to the reported T_max_ of naloxone (NLX).^9^ The heroin‐related death is a remarkable issue that encompasses relevant health, judicial and forensic consequences. Frequently, the final diagnosis is based upon a combination of scene investigation, physical examination of the body, the autopsy, as well as histological and toxicological findings.^10^ So, post‐mortem diagnosis of heroin‐related death, could be an enigma.^11^

Toxicology data is certainly of greater importance for the diagnosis of heroin‐related death. Several implications for research arise from the literature on deaths attributed to heroin overdose because blood morphine alone often cannot explain the fatal event.^12^ A true heroin overdose, in the absence of poly‐drugs abuse, represents a minority of cases, and a more complex mechanism of action, of an inflammatory or immunological nature, has been repeatedly considered.^13^ Lack of tolerance, the synergistic effect of other toxic substances, and even repeated allergic stimuli to adulterants, or to heroin itself, to the extent of inducing anaphylactoid reactions, are theories to be taken seriously.^14^ At present, illicit fentanyl, and other synthetic opioids represent the third wave of the so‐called opioid overdose epidemic.^15^ Research is looking for how vaccines for the treatment of opioid use disorders and reduction of opioid‐induced fatal overdoses fit within the current medication‐assisted treatment portfolio.^16^

The aim of this study was to clarify the correlation between heroin administration and the distributive response in heroin‐related death, as well as to investigate the correlation between heroin blood concentration and the immunohistochemical features of various tissues through an anti‐6‐MAM antibody.

## 2 MATERIAL AND METHODS

The processing of the data reported in this paper is covered by the general authorization to process personal data for scientific research purposes granted by the Italian Data Protection Authority (1 March 2012 as published in Italy's Official Journal no. 72 dated 26 March 2012) since the data do not entail any significant personalized impact on data subjects. Our study does not involve the application of experimental protocols; therefore, it does not require approval by an institutional and/or licensing committee nor informed consent. In all cases, local prosecutors opened an investigation, ordering that an autopsy be performed to clarify the exact cause of death.

### 2.1 Samples selection

The toxicological data and the autopsy records of the 204 autopsies of drug‐related death performed at the Departments of Forensic Pathology of the University of Rome, Foggia and Pisa over the period 2014–2021 were evaluated, and 45 cases of heroin‐related death were selected (42 men, three women, mean age 44.4 years). The autopsy was performed within 36 h after death. Only cases with toxicological data positive for heroin and negative for any other drug (ethanol included) were selected. Post‐mortem examination confirmed the diagnosis of heroin‐related death in all the cases. Toxicological analyses were performed on peripheral blood, bile, and urine by gas chromatography‐mass spectrometry (5977A Series Agilent). The mean concentrations of free‐morphine, total‐morphine, and 6‐monoacetylmorphine are reported in Table 1.

**TABLE 1:** Mean free‐morphine, total‐morphine, and 6‐monoacetylmorphine concentrations in peripheral blood, bile and urine in the 45 cases of heroin‐related death selected from the databases

Substances	Mean concentration (µg/ml)		
	Peripheral Blood	Bile	Urine
Free‐morphine	0.344 C.R. (0.030–1.170)	2.929 C.R. (0.182–10.43)	0.636 C.R. (0.019–3.420)
Total‐morphine	0.508 C.R. (0.102–1.240)	6.990 C.R. (0.081–38.70)	2.281 C.R. (0.060–11.39)
6‐monoacetylmorphine	0.041 C.R. (0–0.152)	0.115 C.R. (0–0.781)	0.357 C.R. (0–1.850)

Note

C.R. indicates maximum and minimum concentration range.

All cases were HIV‐1 negative. The control group was composed of 15 cases (five women, ten men, mean age 43.9 years) that died from car accidents (*n* = 3) and sudden cardiac deaths (*n* = 2) with no clinical history, without brain lesions and negative toxicological analysis for drugs. Those deaths were characterized by their rapidity.

### 2.2 Histological and immunohistochemical analysis

The study was carried out on samples of the brain (superior frontal gyrus and hypothalamus), heart, lung, liver and kidney. In the literature, there were no indications on the best method to be used in immunohistochemistry with the Polyclonal 6‐Monoacetylmorphine; antibody Ig fraction supplied in liquid form in 20 mM phosphate buffer, 150 mM sodium chloride and 0.09% sodium azide, pH 7.2 (Fitzgerald clone: 20–1488. Heroin antibody was raised in sheep using 6‐Monoacetylmorphine CJ‐4–180 as the immunogen; furnished by Labprice, Kampenhout, Belgium). Lacking other similar experimental studies, we had to establish from scratch an adequate method and the appropriate dilution of the antibody.

Samples of each organ from each case were fixed in 10% buffered formalin, then washed with phosphate‐buffered saline (PBS) and subsequent dehydration was carried out using a graded alcohol series. After dehydration, samples were cleared in xylene and embedded in paraffin. Sections were cut at 4 μm, mounted on slides, and covered with 3‐amminopropyltriethoxysilane (Fluka). The sections were pretreated with endogenous peroxidase activity, incubated with the anti‐6‐Monoacetylmorphine antibody diluted 1:100. After removal of the primary antibodies with three 5‐min washes in PBS, sections were incubated for 40 min with biotinylated horse anti‐mouse IgG (Vector) diluted 1:200 in 1% NHS. After three 5‐min washes in PBS, sections were incubated for 30 min with horseradish peroxidase avidin D (HRP, Vector) diluted 1:1000 with PBS. After three 5‐min washes of PBS, the sections were developed with the DAB kit (Vector), stopped with rinses of double‐distilled water.

Routine microscopic histopathological studies were performed using formalin‐fixed paraffin‐embedded tissue sectioned at 4 μm and stained with haematoxylin‐eosin (H & E). The specimens were examined under transmitted bright field illumination and phase contrast mode using a light microscope (DM 4000 B Leica) connected to a photo camera computer system (DC 480 Leica). Three‐dimensional reconstruction of tissues utilizing a confocal microscope (True Confocal Scanner, Leica TCS SP2) was performed.

The immunohistochemical findings and the gradation of the immunohistochemical reaction have been described with an ordinary scale and the median value has been reported. For the quantitative analysis, in each immunohistochemical section, we made 20 observations in different fields/slides. The 6‐Monoacetylmorphine‐stained cells were counted at 60× using a light microscope coupled to a high‐resolution colour video camera. The reactions were graded as follows: 0. (−) not expressed; 1 (+) isolated and disseminated expression; 2 (++) expression in scattered foci; 3 (+++) expression in widespread foci; and 4 (++++) widespread expression. The evaluations were carried out separately for each tissue, using a double‐blind method. In cases of divergent scoring, a third observer decided the final score.

## 3 RESULTS

**FIGURE 1:**
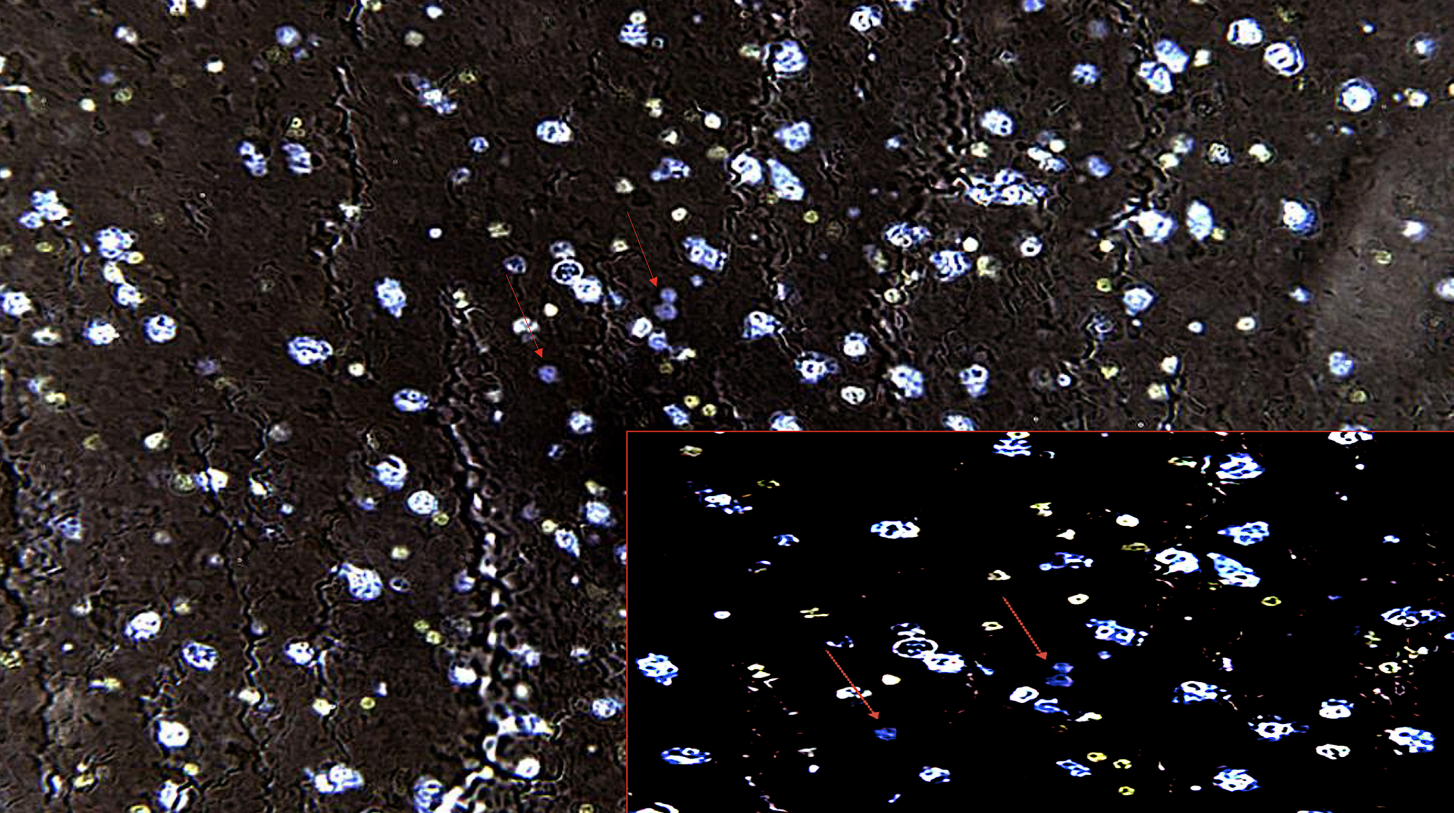
Confocal laser scanning microscopy (CLSM): intense immunoreactivity in the hypothalamus demonstrated by purple spots (arrows) in the parenchyma (neuronal nuclei in white, cytoplasm in bleu) (×100). Insert: ×150

**FIGURE 2:**
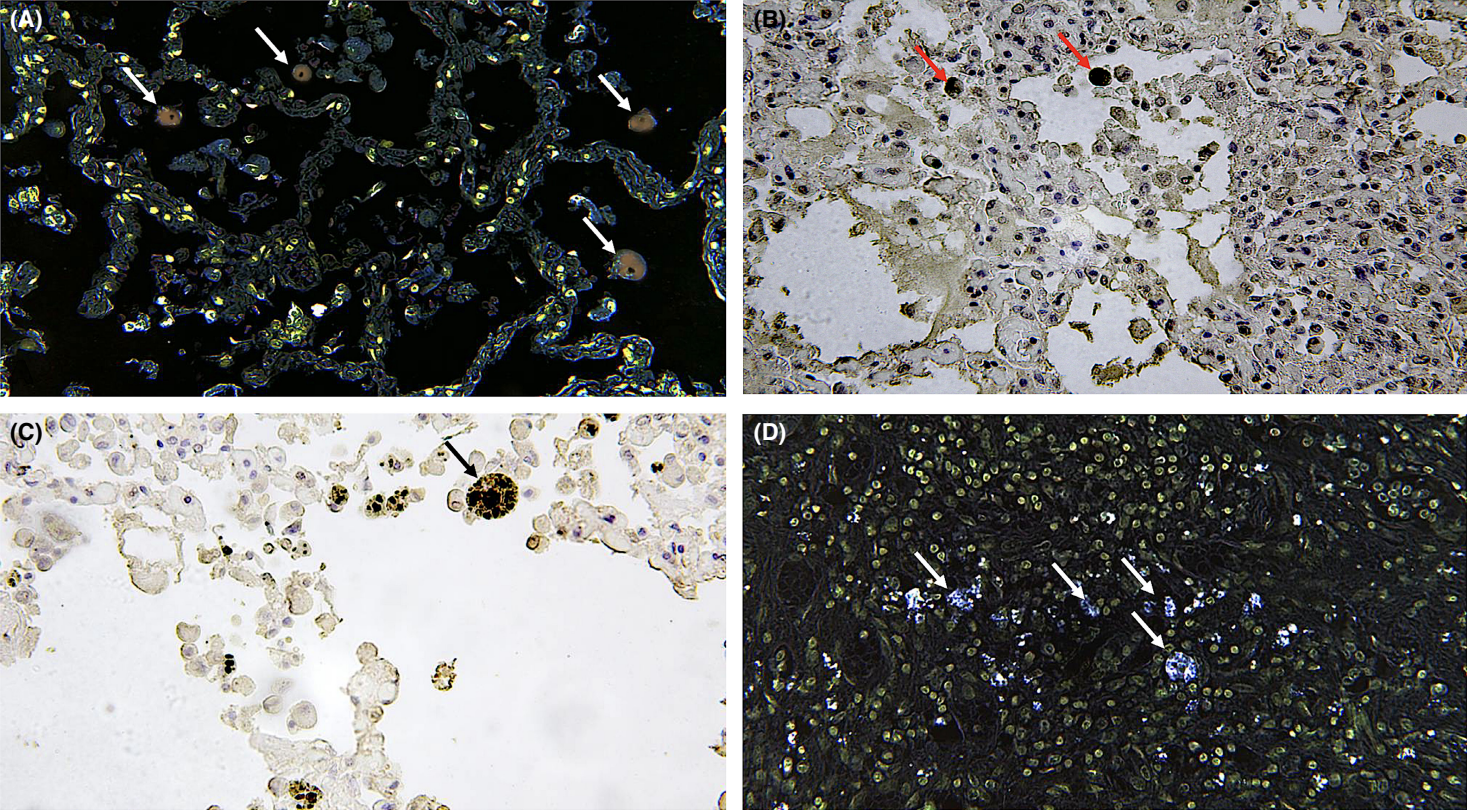
Lung: (A, B) evident staining (arrows) in the cytoplasm of macrophages (CLSM positivity in brown, ×100) and higher magnification (×200) in (C) and (D) spots (in bleu) in the intra‐alveolar spaces (arrows) (CLSM positivity in bleu, ×60)

**FIGURE 3:**
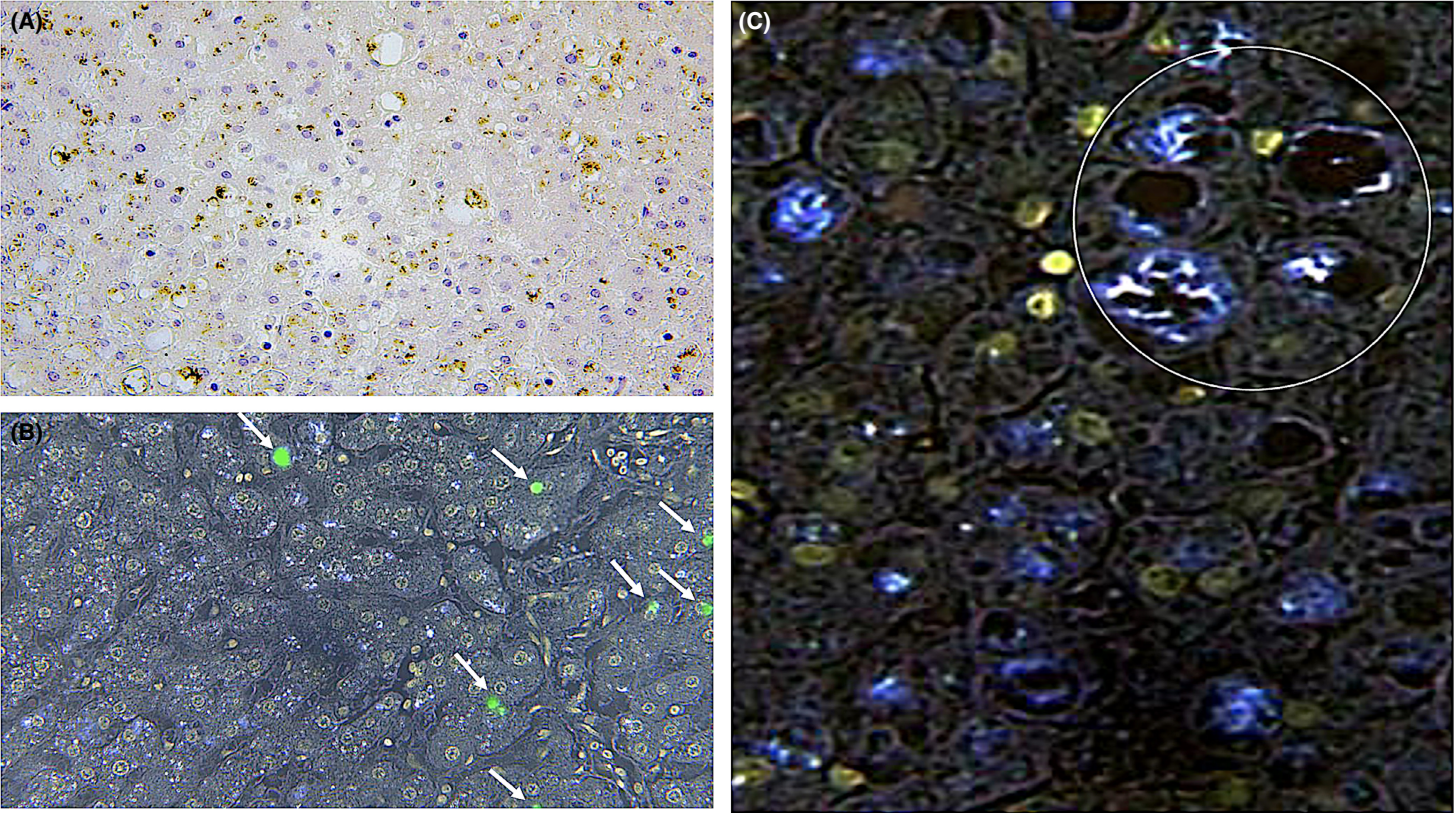
(A) Positivity reactions are evident (in brown, ×60); (B) visualization of focal positivity (arrows) with confocal laser scanning microscope (green spots) (×80). (C) Intense positivity (in bleu, ×200) near a portal space (delimited by white circle)

**FIGURE 4:**
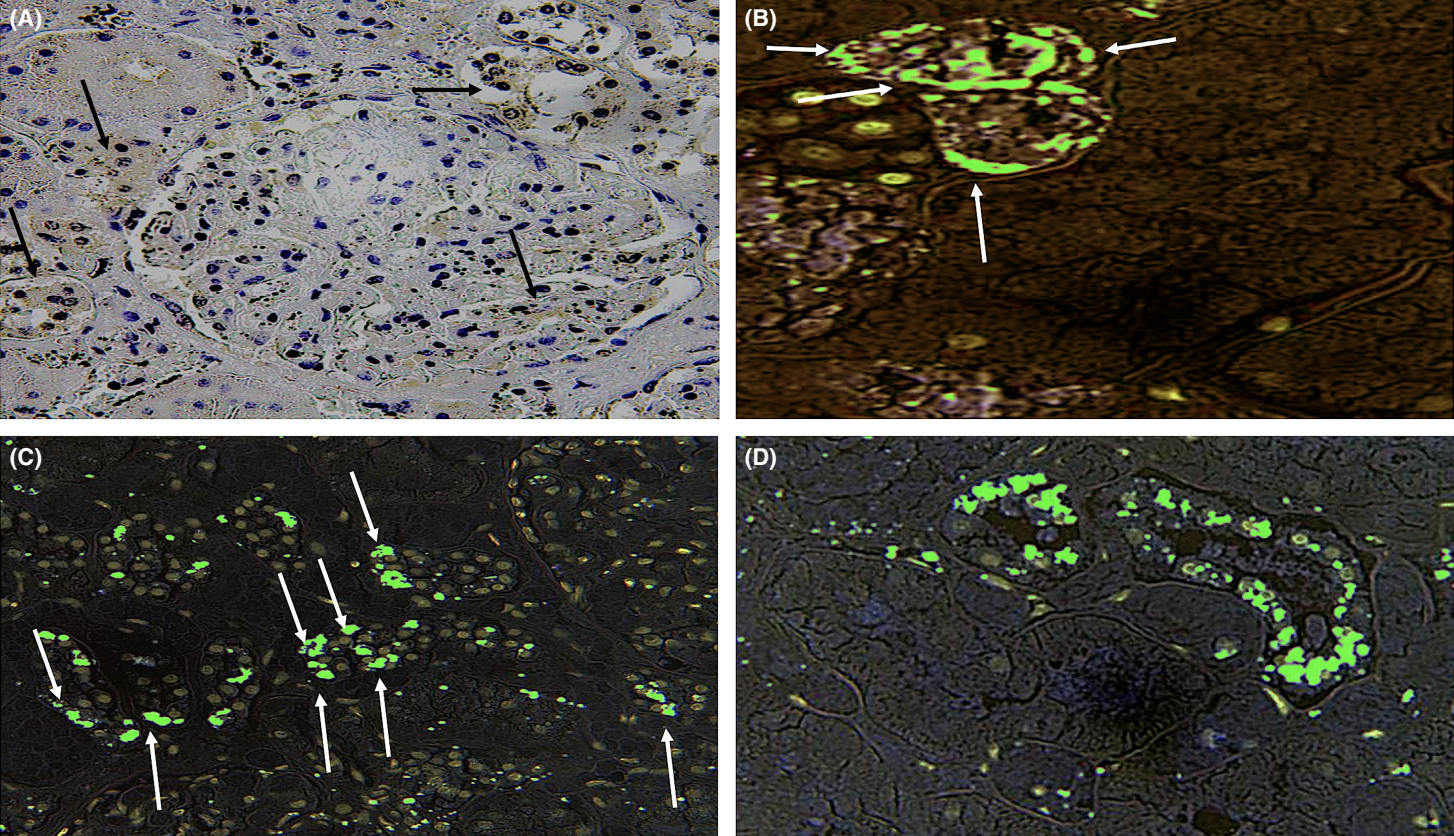
(A) Glomerular positivity (in brown, arrows) and at the level of both proximal and distal convoluted tubules (arrows, ×300). (B–D) Confocal laser scanning microscopy study of the kidneys. (B) Intense positivity (in green) within the tubular epithelial cells in the proximal tubules (arrows, ×300) and (C, D) at the level of both proximal and distal convoluted tubules (arrows, ×200)

**FIGURE 5:**
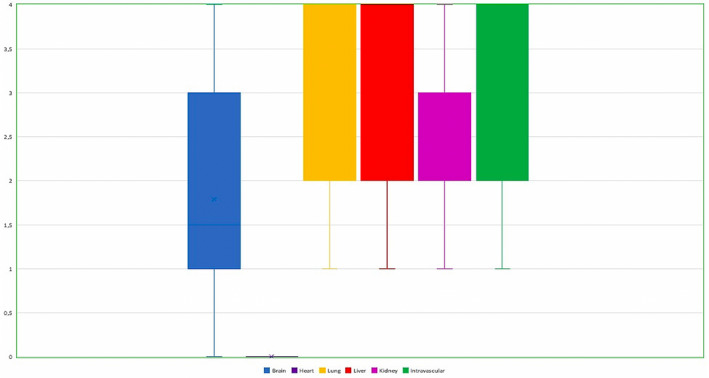
Box and whiskers plot showing the immunohistochemical reaction in the brain, lung, liver, kidney and intravascular

In our study, samples obtained from 45 cases of acute heroin‐related death, with negative toxicological analysis for other drugs, were analysed through immunohistochemistry to evaluate the pattern of heroin distribution in various tissues. The immunohistochemical technique allowed us to quantify the expression of the response, as shown in Table 2. We found blood vessel immunostaining in tissue samples. In brain heroin‐expressing neurons, segmental cell loss in the hypothalamus and ischemic damage to nerve cells, as reflected by cytoplasmic eosinophilia and/or loss of Nissl bodies, were evident in all victims surviving longer (as demonstrated by tox analysis) (Figure 1). The proliferation of heroin‐expressing astrocytes and/or heroin‐expressing microglial cells in the regions of the cortex (semi‐quantitative analysis) was also noticed. A microglial or astroglial reaction, or both, was detected in a major percentage of the cases investigated. In the lung, we found clear staining in the cytoplasm of macrophages and spots in the intra‐alveolar spaces (Figure 2). No anti‐6‐MAM staining was found in myocardial samples. In the liver, the immunostaining appeared in hepatocytes, in the duct epithelium or the portal‐biliary space (Figure 3). In kidney tissue samples, we found immunostaining in the glomeruli and renal tubules. Three‐dimensional images of histological sections on confocal laser scanning microscopy were recorded (Figure 4). A positive reaction was evident on optical microscopy and a 3‐D reconstruction was made by a confocal laser scanning microscopy. Intense immunostaining positivity within the tubular epithelial cells and widespread necrosis of the tubular epithelium in the proximal tubules were seen. Figure 5 shows the greatest immunohistochemical reaction in the brain, lung, liver and kidney.

**TABLE 2:** Semi‐quantitative evaluation and statistical analysis [statistically significant (*p* < 0.05)] of the immunohistochemical findings and gradation of the immunohistochemical reaction in the brain, heart, lung, liver, kidney, intravascular samples in the heroin‐related deaths and in the control group

Groups	Immunohistochemical reaction (anti‐heroin antibody) Semi‐quantitative mean values					
	Brain	Heart	Lung	Liver	Kidney	Intravascular
Heroin‐related deaths (*n* = 45)	1.75	Negative	2.64	2.68	2.48	3.15
Control group (*n *= 15)	Negative	Negative	Negative	Negative	Negative	Negative

The positive reaction in the different organs was related to the timing of heroin metabolization, being most positive in the brain and lungs in the earliest intakes, while the highest positivity in the liver and kidneys was found in cases where the heroin was already metabolized and being excreted. The anti‐6‐MAM antibody exhibited no reaction in organs or blood vessels of cadavers who died from non‐heroin‐related death (control group). Table 3 shows the comparison between the toxicological data and immunohistochemical reactions, as well as the PMI, in our cases.

**TABLE 3:** Comparison between the toxicological and immunohistochemical results, alongside the PMI of each case

Case	Toxicological data (µg/ml)			Immunohistochemical reaction						PMI (hours)
	Peripheral Blood	Bile	Urine	Brain	Heart	Lung	Liver	Kidney	Intravascular	
1	F.M: 0.477 T. M: 0.657 6‐ MAM: 0.043	F.M: 1.74 T. M: 13.02 6‐ MAM: negative	F.M.: 0.214 T. M.: 0.306 6‐ MAM: 0.406	+ +	Negative	+ + +	+	+ + +	+ + + +	25
2	F.M: 0.511 T. M: 0.629 6‐ MAM: 0.076	F.M: 0.98 T. M: 1.54 6‐ MAM: 0.321	F.M.: 0.165 T. M.: 0.754 6‐ MAM: 0.035	+ + + +	Negative	+ + + +	+ + + +	+ +	+ + + +	33
3	F.M: 0.269 T. M: 0.432 6‐ MAM: 0.024	F.M: ‐‐‐ T. M: 8.23 6‐ MAM: ‐‐‐	F.M.: 0.115 T. M.: 0.499 6‐ MAM: 0.065	+	Negative	+	+ + + +	+ +	+ + + +	24
4	F.M: 0.378 T. M: 0.506 6‐ MAM: 0.053	F.M: 5 T. M: 5.98 6‐ MAM: 0.665	F.M.: 0.149 T. M.: 1.53 6‐ MAM: 0.237	+ +	Negative	+ + +	+ + + +	+ + +	+ + + +	26
5	F.M: 0.243 T. M: 0.549 6‐ MAM: 0.064	F.M: 0.182 T. M: 0.708 6‐ MAM: 0.21	F.M.: 5.59 T. M.: 7.24 6‐ MAM: 0.306	+ +	Negative	+ + + +	+ + +	+ + + +	+ + + +	27
6	F.M: 0.218 T. M: 0.402 6‐ MAM: 0.086	F.M: 0.384 T. M: 22.01 6‐ MAM: 0.017	F.M.: 3.42 T. M.: 11.39 6‐ MAM: 1.01	+ + +	Negative	+ + + +	+ + +	+ + + +	+ + + +	25
7	F.M: 0.133 T. M: 0.196 6‐ MAM: ‐‐‐	F.M: 6 T. M: 31.8 6‐ MAM: 0.199	F.M.: 0.111 T. M.: 0.458 6‐ MAM: 0.092	+	Negative	+ + +	+ + + +	+ + +	+ + + +	26
8	F.M: 0.478 T. M: 0.798 6‐ MAM: 0.124	F.M: 0.432 T. M: 0.567 6‐ MAM: 0.045	F.M.: 2.06 T. M.: 4.61 6‐ MAM: 0.288	+ + + +	Negative	+ + + +	+ + +	+ +	+ + + +	36
9	F.M: 0.373 T. M: 0.843 6‐ MAM: 0.047	F.M: 3.19 T. M: 38.7 6‐ MAM: 0.061	F.M.: 0.205 T. M.: 0.721 6‐ MAM: 0.072	+ + +	Negative	+ + + +	+ + +	+ +	+ + + +	32
10	F.M: 0.342 T. M: 0.567 6‐ MAM: 0.025	F.M: ‐‐‐ T. M: 8.67 6‐ MAM: negative	F.M.: 0.35 T. M.: 0.628 6‐ MAM: 0.085	+ + +	Negative	+ + + +	+	+ +	+ + + +	25
11	F.M: 0.429 T. M: 0.525 6‐ MAM: 0.005	F.M: 3.31 T. M: 5.36 6‐ MAM: 0.078	F.M.: 1.69 T. M.: 4.12 6‐ MAM: 0.792	+	Negative	+	+ + +	+ + + +	+ +	24
12	F.M: 0.232 T. M: 0.587 6‐ MAM: 0.053	F.M: ‐‐‐ T. M: 0.822 6‐ MAM:0.088	F.M.: 1.08 T. M.: 2.75 6‐ MAM: 1.11	+ +	Negative	+ + +	+ + +	+ + + +	+ + + +	24
13	F.M: 0.199 T. M: 0. .399 6‐ MAM: 0.049	F.M: 0.19 T. M: 1.33 6‐ MAM: negative	F.M.: 0.213 T. M.: 0.732 6‐ MAM: 0.108	+ +	Negative	+ +	+	+ +	+ + + +	34
14	F.M: 0.12 T. M: 0.204 6‐ MAM: 0.013	F.M: 10.43 T. M: 19.25 6‐ MAM: ‐‐‐	F.M.: 0.14 T. M.: 0.53 6‐ MAM: 0.088	+	Negative	+	+ + + +	+	+ +	26
15	F.M: 0.105 T. M: 0.173 6‐ MAM: 0.016	F.M: 4.56 T. M: 8.22 6‐ MAM: 0.624	F.M.: 0.085 T. M.: 0.432 6‐ MAM: 0. .059	+	Negative	+ +	+ + + +	+ +	+ +	30
16	F.M: 0.283 T. M: 0.311 6‐ MAM: negative	F.M: ‐‐‐ T. M: 2.452 6‐ MAM:‐‐‐	F.M.: 0.114 T. M.: 0.352 6‐ MAM: 0.03	Negative	Negative	+ +	+ + +	+ +	+	30
17	F.M: 0.281 T. M: 0.374 6‐ MAM: 0.09	F.M: 2.15 T. M: 3.43 6‐ MAM: 0.781	F.M.: 2.72 T. M.: 7.46 6‐ MAM: 1.85	+	Negative	+ +	+ + + +	+ + + +	+ + + +	31
18	F.M: 0.786 T. M: 1.03 6‐ MAM: 0.065	F.M: ‐‐‐ T. M: 2.84 6‐ MAM:‐‐‐	F.M.: 0.48 T. M.: 0.79 6‐ MAM: 0.02	+ +	Negative	+ + + +	+ + +	+ +	+ + + +	26
19	F.M: 0.045 T. M: 0.221 6‐ MAM: negative	F.M: 0.78 T. M: 1.31 6‐ MAM: 0.031	F.M.: 0.34 T. M.: 1.9 6‐ MAM: 0.567	Negative	Negative	+	+ +	+ + +	+	24
20	F.M: 0.243 T. M: 0.522 6‐ MAM: 0.053	F.M: 2.92 T. M: 4.07 6‐ MAM: 0.082	F.M.: 0.174 T. M.: 0.93 6‐ MAM: 0.231	+ +	Negative	+ + +	+ + +	+ +	+ + + +	24
21	F.M: 0.154 T. M: 0.238 6‐ MAM: negative	F.M: ‐‐‐ T. M: 3.12 6‐ MAM: 0.059	F.M.: 0.081 T. M.: 0.243 6‐ MAM: 0.09	Negative	Negative	+ +	+ + +	+ +	+	26
22	F.M: 0.399 T. M: 0.654 6‐ MAM: 0.053	F.M: ‐‐‐ T. M: 0.383 6‐ MAM: negative	F.M.: 0.367 T. M.: 3.41 6‐ MAM: negative	+	Negative	+ + +	+	+	+ + + +	27
23	F.M: 0.479 T. M: 0.795 6‐ MAM: 0.084	F.M: 2.77 T. M: 5.78 6‐ MAM: ‐‐‐	F.M.: 1.03 T. M.: 2.52 6‐ MAM: 0.387	+ + +	Negative	+ + + +	+ + +	+ +	+ + + +	30
24	F.M: 0.655 T. M: 0.943 6‐ MAM: 0.038	F.M: 0.182 T. M: 0.708 6‐ MAM: 0.038	F.M.: 0.456 T. M.: 1.39 6‐ MAM: 0.157	+ +	Negative	+ + + +	+ +	+ +	+ + + +	36
25	F.M: 0.215 T. M: 0.274 6‐ MAM: 0.018	F.M: ‐‐‐ T. M: 0.312 6‐ MAM:‐‐‐	F.M.: 0.126 T. M.: 0.432 6‐ MAM: 0.065	+	Negative	+ +	+ +	+ +	+ + + +	36
26	F.M: 0.208 T. M: 0.244 6‐ MAM: negative	F.M: ‐‐‐ T. M: 2.13 6‐ MAM: negative	F.M.: 0.16 T. M.: 0.567 6‐ MAM: 0.124	Negative	Negative	+	+	+ + +	+	34
27	F.M: 0.03 T. M: 0.102 6‐ MAM: negative	F.M: 10.11 T. M: 17.22 6‐ MAM: negative	F.M.: 0.28 T. M.: 1.31 6‐ MAM: 0.893	Negative	Negative	+	+	+ + +	+	24
28	F.M: 0.195 T. M: 0.281 6‐ MAM: 0.005	F.M: ‐‐‐ T. M: 0.081 6‐ MAM: negative	F.M.: 0.04 T. M.: 0.09 6‐ MAM: negative	+	Negative	+ +	+	+	+ +	25
29	F.M: 0.207 T. M: 0.276 6‐ MAM: 0.015	F.M: 2.71 T. M: 3.76 6‐ MAM: 0.187	F.M.: 0.088 T. M.: 0.238 6‐ MAM: 0.098	+	Negative	+ +	+ + + +	+ +	+ + +	25
30	F.M: 1.17 T. M: 1.24 6‐ MAM: 0.115	F.M: 1.72 T. M: 2.98 6‐ MAM: ‐‐‐	F.M.: 1.05 T. M.: 2.21 6‐ MAM: 0.927	+ +	Negative	+ + +	+ +	+ + + +	+ + + +	30
31	F.M: 0.538 T. M: 0.732 6‐ MAM: 0.102	F.M: 4.67 T. M: 7.47 6‐ MAM: 0.08	F.M.: 0.295 T. M.: 2.88 6‐ MAM: 1.17	+ + + +	Negative	+ + + +	+ +	+ + + +	+ + + +	31
32	F.M: 0.287 T. M: 0.391 6‐ MAM: 0.007	F.M: 5.11 T. M: 7.01 6‐ MAM: 0.021	F.M.: 0.056 T. M.: 0.632 6‐ MAM: 0.276	+	Negative	+ +	+ +	+ +	+ +	27
33	F.M: 0.166 T. M: 0.187 6‐ MAM: 0.009	F.M: 2.8 T. M: 19.24 6‐ MAM: 0.069	F.M.: 2.43 T. M.: 16.18 6‐ MAM: 0.179	+	Negative	+	+ + + +	+ +	+ +	28
34	F.M: 0.209 T. M: 0.284 6‐ MAM: 0.023	F.M: ‐‐‐ T. M: 3.81 6‐ MAM: ‐‐‐	F.M.: 0.403 T. M.: 0.983 6‐ MAM: 0.522	+	Negative	+ +	+ + +	+ + +	+ + + +	25
35	F.M: 0.568 T. M: 0.798 6‐ MAM: 0.140	F.M: 0.97 T. M: 1.47 6‐ MAM: 0.043	F.M.: 0.102 T. M.: 4.83 6‐ MAM: 0.367	+ + + +	Negative	+ + + +	+ + +	+ + +	+ + + +	30
36	F.M: 0.125 T. M: 0.253 6‐ MAM: negative	F.M: 4.122 T. M: 8.55 6‐ MAM: 0.092	F.M.: 0.149 T. M.: 0.724 6‐ MAM: 0.412	Negative	Negative	+	+ + + +	+ + +	+	33
37	F.M: 0.379 T. M: 0.921 6‐ MAM: 0.068	F.M: 3.2 T. M: 9.7 6‐ MAM:0.082	F.M.: 0.085 T. M.: 0.843 6‐ MAM: 0.498	+ + +	Negative	+ + + +	+ + + +	+ + +	+ + + +	32
38	F.M: 0.417 T. M: 0.484 6‐ MAM: 0.059	F.M: 4.98 T. M: 6.71 6‐ MAM: 0.079	F.M.: 0.06 T. M.: 0.294 6‐ MAM: 0.125	+ +	Negative	+ + + +	+ + +	+ +	+ + + +	27
39	F.M: 0.379 T. M: 0.567 6‐ MAM: 0.102	F.M: ‐‐‐ T. M: 5.82 6‐ MAM: ‐‐‐	F.M.: 0.32 T. M.: 1.12 6‐ MAM: 0.876	+ + + +	Negative	+ + + +	+ + + +	+ + + +	+ + + +	28
40	F.M: 0. .278 T. M: 0.324 6‐ MAM: 0.004	F.M: ‐‐‐ T. M: 1.02 6‐ MAM: ‐‐‐	F.M.: 0.079 T. M.: 0.269 6‐ MAM: 0.095	+ +	Negative	+ +	+ + +	+ +	+ +	31
41	F.M: 1.05 T. M: 1.17 6‐ MAM: 0.027	F.M: 0.64 T. M: 0.97 6‐ MAM: 0.015	F.M.: 0. .031 T. M.: 0.107 6‐ MAM: 0.032	+ +	Negative	+ + +	+ + +	+ +	+ + + +	29
42	F.M: 0.248 T. M: 0.291 6‐ MAM: 0. .032	F.M: 0.25 T. M: 1.59 6‐ MAM:negative	F.M.: 0.019 T. M.: 0.06 6‐ MAM:negative	+	Negative	+ +	+	+	+ + + +	25
43	F.M: 0.282 T. M: 0.357 6‐ MAM: 0.008	F.M: 1.82 T. M:2.32 6‐ MAM:negative	F.M.: 0.066 T. M.: 0.193 6‐ MAM: 0.087	+ +	Negative	+ +	+	+	+ +	25
44	F.M: 0.111 T. M: 0.322 6‐ MAM: negative	F.M: ‐‐‐ T. M: 5.71 6‐ MAM: 0. .051	F.M.: 0.504 T. M.: 0.868 6‐ MAM: 0.243	Negative	Negative	+	+ + +	+ +	+	27
45	F.M: 0.588 T. M: 0.799 6‐ MAM: 0.152	F.M: ‐‐‐ T. M: 16.39 6‐ MAM: negative	F.M.: 0.932 T. M.: 9.37 6‐ MAM: 0.984	+ + + +	Negative	+ + + +	+	+ + + +	+ + + +	30

Abbreviations: 6‐ MAM, 6‐monoacetylmorphine; F.M., indicates free‐morphine; PMI, post‐mortem interval; T.M., total‐morphine.

## 4 DISCUSSION

We used an experimental model and immunohistochemistry to investigate the post‐mortem anti‐6‐MAM antibody expression and localization in heroin‐relation death cases. Our preliminary study's objective was to find out if immunohistochemistry, using an anti‐6‐MAM antibody, could represent an effective means to diagnose acute heroin abuse. In brain tissue, extensive 6‐MAM‐reaction positivity and microglial or astroglial reaction, or both, were detected in all the investigated cases.^17^ The positive heroin reactions in the frontal cortex and hypothalamus, as expected based on the mechanism of action in humans, are excretion‐related and could provide interesting data about the timing of consumption of the drug.^18^, ^19^ Our findings on liver and kidney samples could be related to the pathway of excretion of heroin. The elimination pathway for a heroin dose is 80% excreted in the urine, while the liver excretion would be of lesser importance (20%).^20^, ^21^ It is therefore evident that anti‐6‐MAM antibody positivity in kidney tubules, hepatocytes and liver ducts would represent a normal physiological finding related to the elimination of metabolites of the heroin from blood circulation, and, in fact, an indirect finding of the elevated heroin concentration.

Additionally, our findings on lung samples showed positivity in the cytoplasm of macrophages and intra‐alveolar spaces can be correlated with acute respiratory depression heroin‐related death.^22^ Macroscopic and microscopic pathological findings in case of heroin‐related death are nonspecific and may be inadequate to render a definitive diagnosis for forensic purposes.^23^ Autopsies in such cases typically show severe pulmonary congestion and oedema weighing >1000 g per lung with a dilated right ventricle.^24^ In cases of chronic and, in particular, intravenous drug consumption, histopathological findings are described, nonspecific, in all organs. One of the causes of death in heroin addiction is respiratory failure, often accompanied by pulmonary complications, especially oedema. The death is generally due to severe acute poisoning, regarding the degree of opioid tolerance possessed by the subjects at the time of the lethal dose.

As Büttner stated, up to 90% of all cases of heroin‐related death show brain oedema with prominent tonsillar herniation and uncal grooving at autopsy.^25^ However, rapid death after heroin intake has no, or poor, morphological evidence of cell injury. Heroin‐related death can occur from the following: absolute acute poisoning; intake of a quantity of heroin superior, in an absolute sense, to any tolerance; acute intoxication related to tolerance: intake of a quantity of heroin greater than the degree of tolerance of the subject; first injection death: taking a lethal dose in a non‐drug addict.^26^

Katz et al. in 1972 highlighted the role of heroin in increasing pulmonary alveolocapillary permeability and together with a loss of albumins in the oedema fluid.^27^ Subsequently Smith et al. showed, in subjects with many years of intravenous drug consumption, deposits of immunoglobulin and complement detected in the pulmonary interstitium.^28^, ^29^ Acute bacterial and nonbacterial endocarditis in intravenous drug‐related deaths can be observed in the myocardium.^30^, ^31^

In the kidneys, related microscopic changes to the use of heroin are defined as ‘heroin‐associated nephropathy’.^32^ The spectrum of heroin‐associated kidney diseases includes primarily acute kidney failure, glomerulopathies, such as focal segmental glomerulosclerosis, membranoproliferative glomerulonephritis, and less often with immune complex glomerulonephritis.^33^ A further histological report is rhabdomyolysis after heroin consumption.^34^ On the central nervous system, heroin has various effects including hypoxic‐ischemic brain damage from respiratory depressing effects and neuroinflammatory response.^25^, ^35^ In an immunohistochemical study, Neri et al. investigated the expression of brain numerous markers.^36^ This study demonstrated that morphine induces inflammatory response and some cytokines were over‐expressed (oxygen‐regulated protein 150, cyclooxygenase‐2, heat shock protein 70, IL‐6 and IL‐15). In another immunohistochemical study, Fineschi et al. confirmed that elevated concentrations of serum tryptase are associated with many heroin‐related deaths.^37^ In several other studies, the morphological and histopathological organs’ changes of chronic heroin abuse are described.^38^, ^39^ These techniques proved to be extremely useful, with very encouraging results, for a diagnosis of chronic heroin abuse, but we still do not have a well‐defined indicator capable of driving a histological and immunohistochemical diagnosis of acute heroin abuse. Cingolani et al. have shown that morphine and its glucuronides are not degraded by formalin, and tissues that have been preserved in formalin can still be analysed for morphine.^40^

One limitation of our study is the relatively small sample size. Precise criteria, however, were used for selecting subjects. Also, detailed immunohistochemical anti‐6‐MAM studies are not available, which precluded a comparison of our observations with others. However, it is important to highlight that this study opens new perspectives in the post‐mortem diagnosis of heroin‐related death, introducing a new, promising diagnostic tool, especially when toxicological data are not available. The results of our immunohistochemical study have shown a good correlation with the toxicological data. Moreover, elucidating the timing of heroin's metabolism may influence therapeutic strategies.^41^

In conclusion, our findings show that immunohistochemistry with the anti‐6‐MAM antibody can be a valuable aid for the post‐mortem diagnosis of acute heroin abuse. The specimens were examined by confocal laser scanning microscopy^42^; we observed an intense positivity corresponding to the tubular cell epithelium and some of them were seen inside the tubular cells. These findings may be of interest to better understand the timing of heroin's metabolism, which can be useful in the forensic field and for future therapeutic applications.^43^

## AUTHOR CONTRIBUTIONS

**Aniello Maiese:** Conceptualization (equal); Formal analysis (equal); Writing; Validation (equal). **Raffaele La Russa:** Methodology; Formal analysis (equal). **Maria Chiara David:** Data curation (equal). **Santina Cantatore:** Data curation (equal); Software. **Alice Chiara Manetti:** Writing – original draft (equal). **Alessandra De Matteis:** Writing – original draft (equal). **Costantino Ciallella:** Writing – review & editing (equal). **Paola Frati:** Validation (equal); Writing – review & editing (equal). **Vittorio Fineschi:** Conceptualization (equal); Supervision; Project administration.

## CONFLICT OF INTEREST

The authors confirm that there are no conflicts of interest.

## Data Availability

The data that support the findings of this study are available from the corresponding author upon reasonable request.
